# Dataset: Compositional analysis and hydrothermal liquefaction of a high-ash microalgae biofilm

**DOI:** 10.1016/j.dib.2025.111490

**Published:** 2025-03-19

**Authors:** Jacob D. Watkins, Hamza Abdellaoui, Elise Barton, Clayton Lords, Ronald C. Sims

**Affiliations:** Utah State University Department of Biological Engineering, 4105 Old Main Hill, RM 402E, Logan, UT, 84322-4105, United States

**Keywords:** Biocrude, Rotating algae biofilm reactor, Proximate analysis, Biochemical analysis, Elemental analysis, Ultimate analysis

## Abstract

This dataset contains biochemical composition data and hydrothermal liquefaction (HTL) yield results for a high-ash microalgae biofilm which was cultivated in effluent from a mesophilic anaerobic digester using polyethylene rotating algae biofilm reactors (RABRs). These data were originally collected for use in a techno-economic analysis of biocrude, biodiesel, and bioplastic production from algae that was cultivated using RABRs for municipal wastewater reclamation.

Biochemical data for the microalgae biomass includes bulk protein, measured both using the Bradford protein assay and by multiplying total N; carbohydrate content, measured using a 3-methyl-2-benzothiazolinone hydrazone / dithiothreitol (MBTH/DTT) assay; total lipid content, measured using a sulpho-phospho-vanillin method; hexane-extractable lipid content, measured by mass difference after extraction with methanol and hexane; ash content, measured by mass difference after incineration at 550°C; moisture content of the harvested biofilm slurry, measured by mass difference after drying at 60°C, mineral composition, measured using an inductively-coupled plasma spectrophotometer; higher heating value, measured using a bomb calorimeter; and CHNS-O elemental composition, measured using an elemental analyser.

Data reported for the HTL product phases include mass yields for each phase (solid, aqueous, biocrude, gas); higher heating value of the biocrude phase, measured using a bomb calorimeter; elemental composition of the biocrude phase, measured using an elemental analyzer; and chemical properties of the aqueous phase, including pH, chemical oxygen demand (HACH method 8000), total nitrogen (HACH method 10,208), total ammonia (HACH method 10,301), total phosphorus (HACH method 10,209/10,210), and total organic carbon (HACH method 10,267).

Currently, the effects of ash composition and HTL heating rate on biocrude yields and on N and P partitioning into biocrude, aqueous, and solid phases are not clearly defined. Models used to predict biocrude yields after HTL of microalgae are commonly trained using data collected from numerous studies. This dataset contains the feedstock composition data and ramp rate data necessary to help define the effects of ash content on biocrude yields after HTL and can be reused to help train yield-prediction models for the HTL of microalgae and other feedstocks.

Specifications Table

**Table:** 

Subject	Bioenergy
Specific subject area	Hydrothermal liquefaction of a high-ash microalgae biofilm
Data format	Raw, Analyzed
Type of data	Tables
Data collection	Data on elemental composition of biomass and HTL products were obtained by inductively-coupled plasma spectrophotometry using a Thermo Electron iCAP ICP (Thermo Scientific Inc, Waltham, MA, USA) and by ultimate analysis using a Flash 2000 CHNS-O analyzer (Thermo Scientific Inc, Waltham, MA, USA). Data on the higher heating value of biomass and HTL products were obtained by bomb calorimetry using an IKA Model C2000 basic bomb calorimeter (IKA Works, Inc., Wilmington, NC, USA).
Data source location	College of Engineering, Department of Biological Engineering, Utah State University, 620 E 1600 N, North Logan, UT, 84341
Data accessibility	Repository name: Mendeley Data Data identification number: 10.17632/fjfkhmn24k Direct URL to data: https://data.mendeley.com/datasets/fjfkhmn24k
Related research article	[1]

## 1 Value of the Data

• This dataset contains microalgae mineral composition data and reports heating ramp rates, both of which are not always reported in microalgae hydrothermal liquefaction studies.
• This dataset can help determine the extents to which ash composition and heating rate affect biocrude yield and HTL process water quality.
• This dataset can be used to help train yield-predictive models for the hydrothermal liquefaction of microalgae biomass.

## 2 Background

This dataset was collected for use in a techno-economic analysis (TEA) study evaluating the economic and technical feasibility of biocrude and bioplastic production from a microalgae biofilm used for nutrient recovery from municipal anaerobic digester centrate [1,2]. HTL testing was performed to estimate biocrude yields for the feedstock assessed in the study and to determine which temperature to use when building process models for the TEA. Characterization of the HTL aqueous phase was performed to determine treatment requirements for the aqueous recycle stream. Quantification of protein, lipid, carbohydrate, and ash content was necessary prior to testing bioplastic production to ensure that the microalgae feedstock would not harm the equipment used in bioplastic production tests. Quantification of the extractable lipid portion was performed to assess the feasibility of co-producing lipid and bioplastic products from the microalgae feedstock. Characterization of heavy metals and minerals was performed to ensure that the final compostable bioplastic product and the solid phase leftover after hydrothermal liquefaction (HTL) of the microalgae biomass did not exceed land application toxicity limits set by the United States Environmental Protection Agency.

## 3 Data Description

The dataset [3] documents the biochemical and elemental composition of a microalgae biofilm community cultured in untreated anaerobic digester centrate using polyethylene Rotating Algae Biofilm Reactors (RABRs), along with product yields measured after hydrothermal liquefaction (HTL) of the same microalgae biofilm samples. The elemental composition of the dataset includes an analysis of heavy metals and trace elements in the biofilm and biochar. The dataset is provided in an Excel spreadsheet on separate sheets. The contents of the dataset are outlined in Table 1 and summarized below.

**Table 1:** Contents of the dataset.

Sheet number	Sheet	Contents
1	Contents	Table of Contents
2	Ultimate_analysis	CHNS data for microalgae biofilm samples and biocrude produced after hydrothermal liquefaction of the biofilm samples
3	ICPS_analysis	Al, As, B, Ba, Ca, Cd, Co, Cr, Cu, Fe, K, Mg, Mn, Mo, Na, Ni, P, Pb, S, Se, Si, Sr, and Zn data for microalgae biofilm samples and biochar samples produced after hydrothermal liquefaction of the biofilm samples
4	HTL_product_yields	Mass in biocrude, solid, and gaseous phases after hydrothermal liquefaction of the biofilm samples
5	Aqueous_phase_analysis	Total nitrogen, total ammonia, total organic carbon, pH, and chemical oxygen demand measured in aqueous phase after hydrothermal liquefaction of the biofilm samples
6	Biochemical_analysis	Protein, lipid, carbohydrate, ash, HHV, and moisture content of the biofilm samples, ash content and HHV of biocrude samples

Biomass samples and products collected after HTL reactions are labelled using the following notation: [biomass source] [HTL product phase] [HTL reaction temperature], where biomass cultivated using either a laboratory RABR (L), pilot RABR (P), or harvested directly from trickling filters at Central Valley Water Reclamation Facility (T); phase is either biomass, biocrude, biochar, gas, or aqueous (Aqueous Phase, AP), and reaction temperature is either 280°C or 350°C. For example, biocrude collected after HTL of biomass cultivated using a laboratory RABR is labelled “L_biocrude_280” for an HTL reaction temperature of 280°C and “L_biocrude_350” for an HTL reaction temperature of 350°C. For biomass samples that have not been subject to an HTL reaction, HTL reaction temperature is not applicable and samples are simply labelled “L_biomass,” “P_biomass,” or “T_biomass”. These abbreviations and their definitions are listed in Table 2 and on a sheet labelled “Contents” in the dataset file.

**Table 2:** Abbreviations used to label samples within the dataset and throughout this document.

Term	Definition
L_biomass	Biomass harvested from a laboratory-scale Rotating Algae Biofilm Reactor (10-L volume)
P_biomass	Biomass harvested from a pilot-scale Rotating Algae Biofilm Reactor (11,400-L volume)
T_biomass	Biomass harvested from trickling filters at Central Valley Water Reclamation Facility in Salt Lake City, Utah
L_biocrude_280	Biocrude produced by hydrothermal liquefaction of L_biomass at 280°C
L_biocrude_350	Biocrude produced by hydrothermal liquefaction of L_biomass at 350°C
P_biocrude_280	Biocrude produced by hydrothermal liquefaction of P_biomass at 280°C
P_biocrude_350	Biocrude produced by hydrothermal liquefaction of P_biomass at 350°C
L_biochar_280	Solid products produced by hydrothermal liquefaction of L_biomass at 280°C
L_biochar_350	Solid products produced by hydrothermal liquefaction of L_biomass at 350°C
P_biochar_280	Solid products produced by hydrothermal liquefaction of P_biomass at 280°C
P_biochar_350	Solid products produced by hydrothermal liquefaction of P_biomass at 350°C
L_AP_280	Aqueous products produced by hydrothermal liquefaction of L_biomass at 280°C
L_AP_350	Aqueous products produced by hydrothermal liquefaction of L_biomass at 350°C
P_AP_280	Aqueous products produced by hydrothermal liquefaction of P_biomass at 280°C
P_AP_350	Aqueous products produced by hydrothermal liquefaction of P_biomass at 350°C

**Fig. 1:**
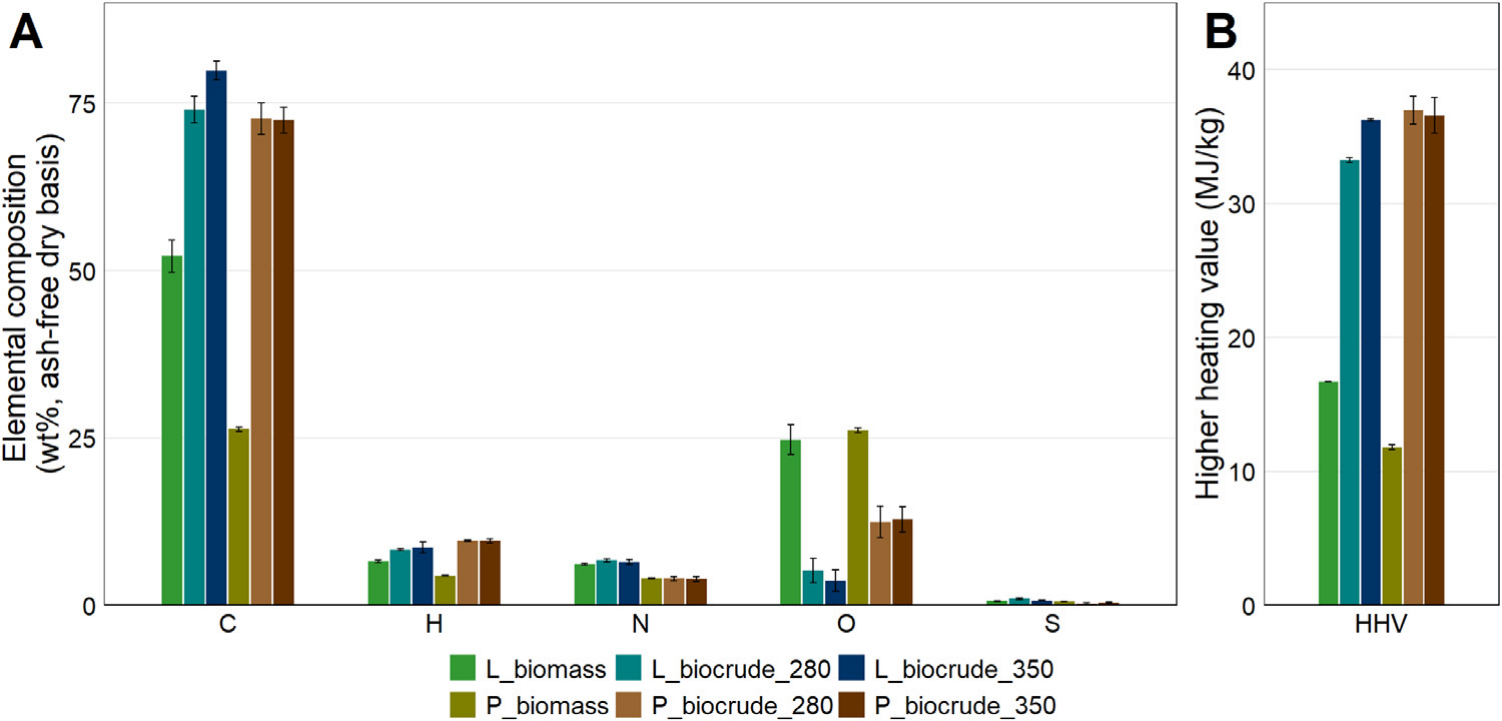
Elemental composition (A) and higher heating value (B) of the lab (L_biomass) and pilot (P_biomass) biofilm samples and of the biocrude samples collected after hydrothermal liquefaction of the same biofilm samples at 280°C and 350°C.

Elemental compositions data (CHNS-O) and higher heating values for biomass and biocrude samples are summarized in Table 3 and Fig. 1. Elemental compositions data is provided in the sheet labelled “Ultimate_analysis” in the dataset file. This sheet includes CHNS data measured by ultimate analysis, and the summary table additionally includes oxygen content, calculated by mass difference, and higher heating values. Higher heating value data are provided in the sheet labelled “Biochemical_analysis”.

**Table 3:** Elemental composition (CHNS-O) and higher heating value (HHV) of biofilm samples and biocrude samples collected after hydrothermal liquefaction at 280°C and 350°C.

	Nitrogen	Carbon	Hydrogen	Sulfur	Oxygen*	HHV (MJ/kg)
L_biomass	6.12 ± 0.15	52.2 ± 2.43	6.51 ± 0.18	0.63 ± 0.07	24.7 ± 2.21	16.7
L_biocrude_280	6.70 ± 0.20	74.1 ± 2.03	8.31 ± 0.13	1.01 ± 0.13	5.19 ± 1.80	33.3 ± 0.17
L_biocrude_350	6.46 ± 0.34	79.9 ± 1.43	8.64 ± 0.79	0.70 ± 0.05	3.69 ± 1.57	36.3 ± 0.06
P_biomass	4.01 ± 0.06	26.3 ± 0.29	4.41 ± 0.07	0.54 ± 0.01	26.2 ± 0.39	11.8 ± 0.20
P_biocrude_280	3.89 ± 0.35	72.4 ± 1.97	9.60 ± 0.33	0.37 ± 0.06	12.8 ± 1.89	36.6 ± 1.34
P_biocrude_350	3.97 ± 0.29	72.7 ± 2.34	9.62 ± 0.09	0.24 ± 0.10	12.4 ± 2.36	37.0 ± 1.04

⁎ by mass difference

**Fig. 2:**
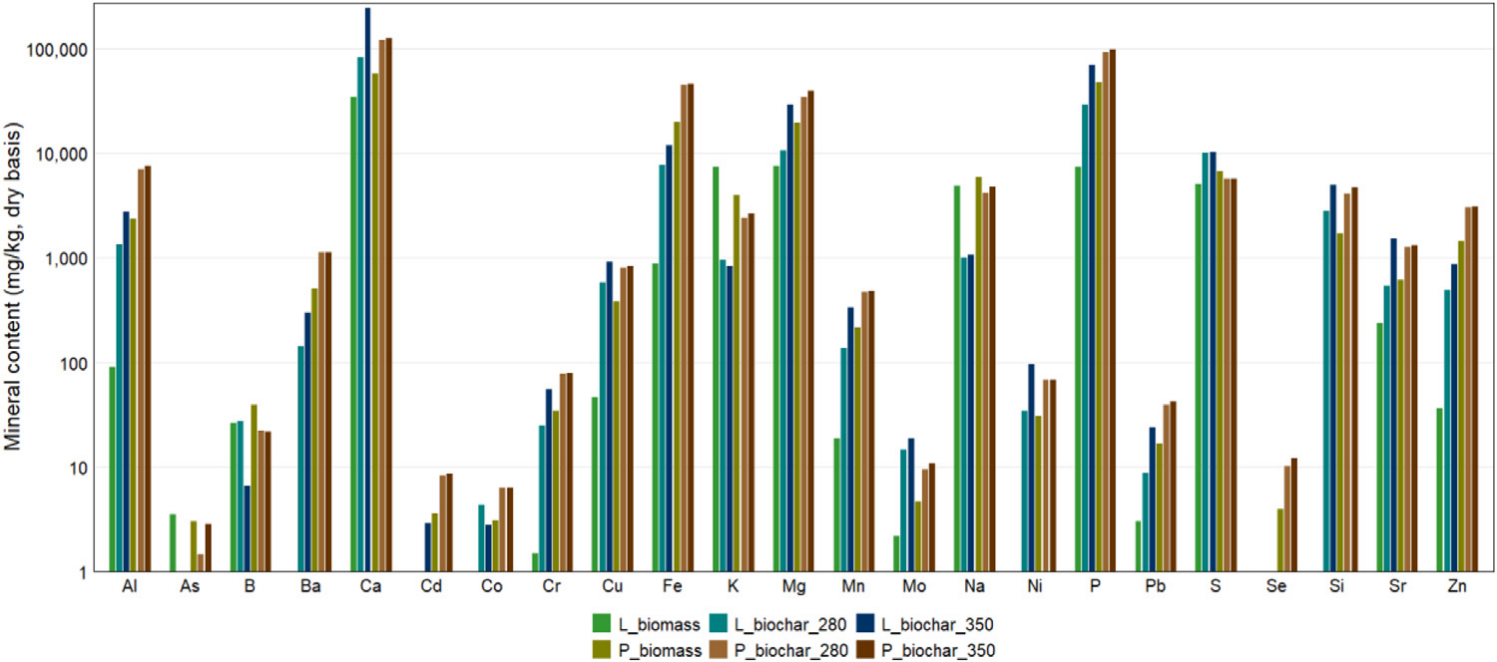
Mineral content of the lab (L_biomass) and pilot (P_biomass) biofilm samples and of the biochar samples collected after hydrothermal liquefaction of the same biofilm samples at 280°C and 350°C.

**Fig. 3:**
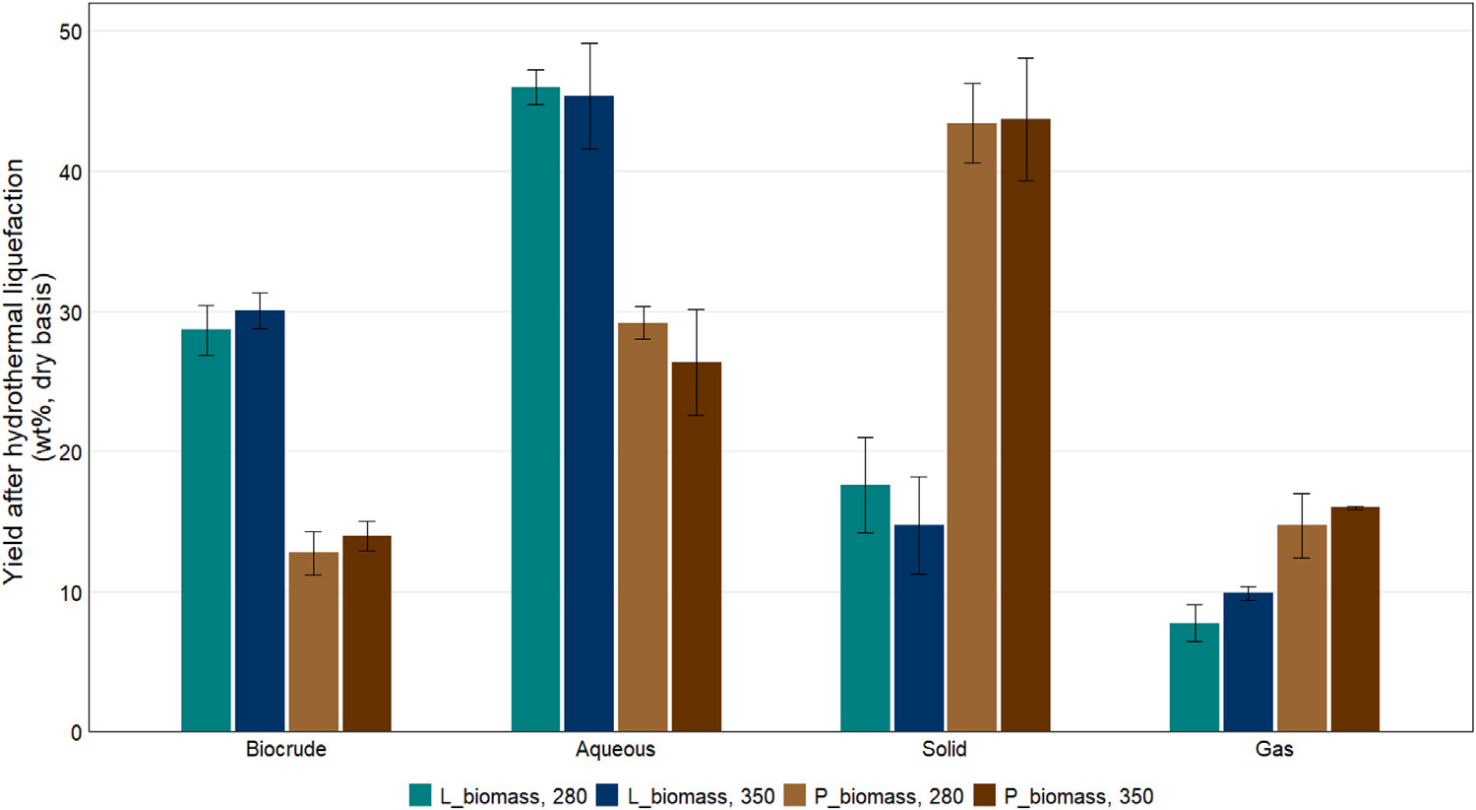
Yield in biocrude, aqueous, solid, and gas phases after hydrothermal liquefaction of lab (L_biomass) and pilot (P_biomass) biofilm samples at 280°C and 350°C.

**Fig. 4:**
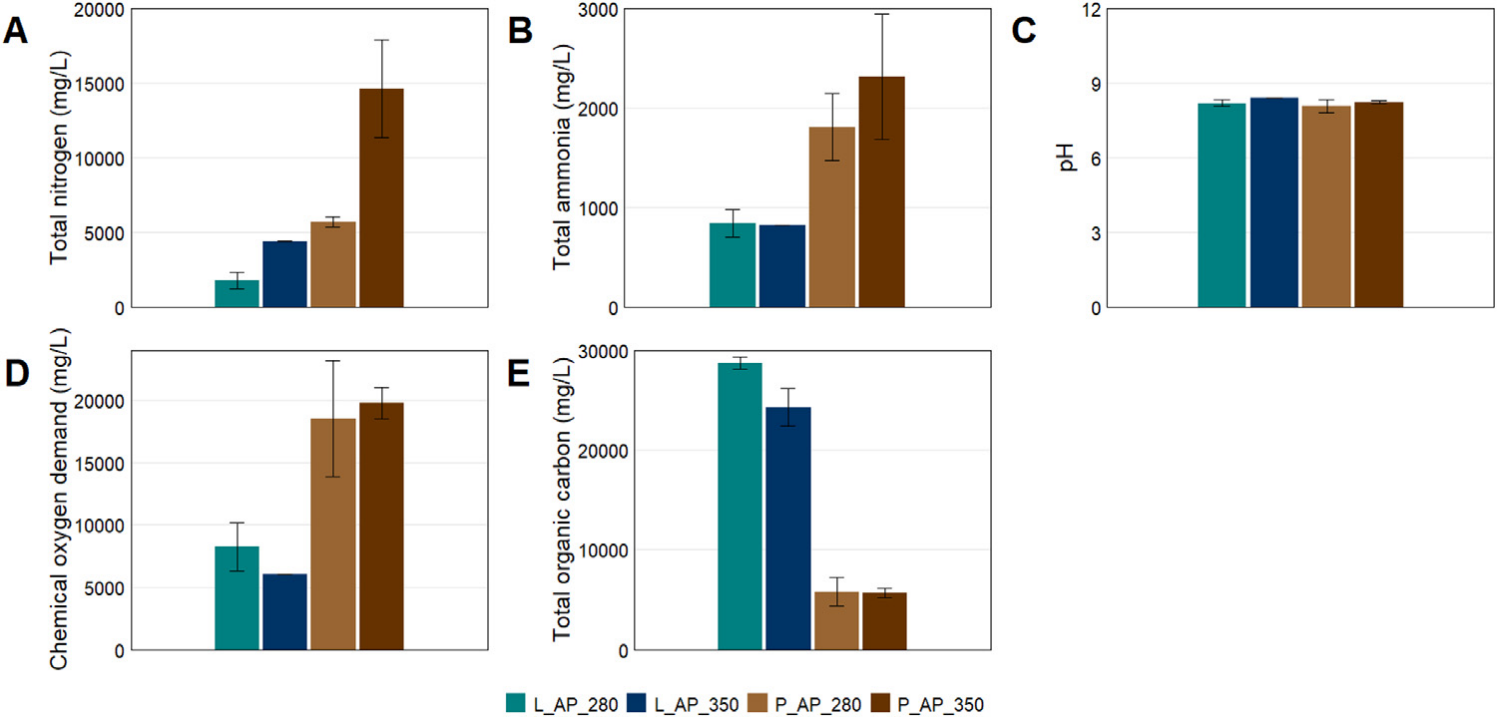
Total nitrogen (A), total ammonia (B), pH (C), chemical oxygen demand (D), and total organic carbon (E) of aqueous products obtained after hydrothermal liquefaction of lab (L) and pilot (P) biofilm samples at 280°C and 350°C.

**Fig. 5:**
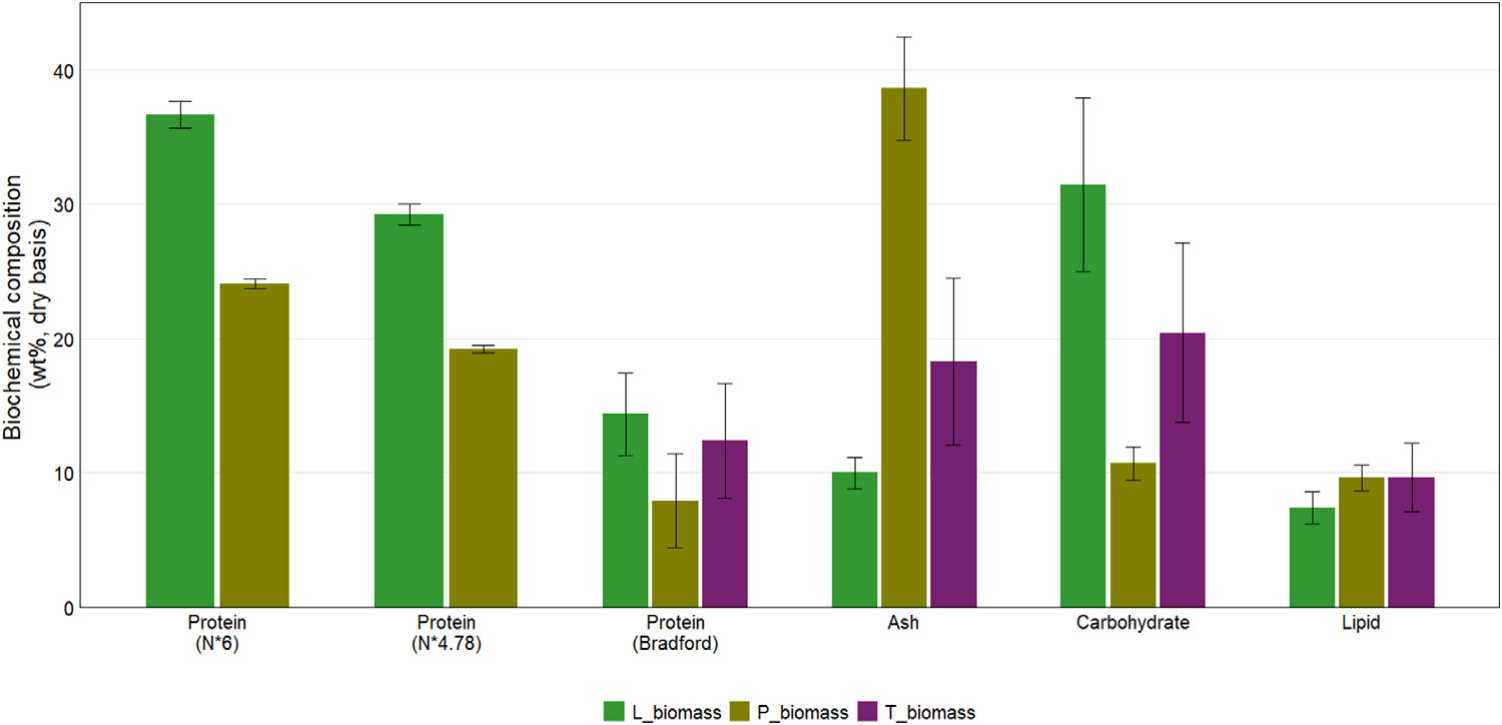
Measured biochemical composition of the lab (L_biomass), pilot (P_biomass), and trickling filter (T_biomass) biofilm samples.

Mineral compositions data for biofilm samples and biochar samples, including Al, As, B, Ba, Ca, Cd, Co, Cr, Cu, Fe, K, Mg, Mn, Mo, Na, Ni, P, Pb, S, Se, Si, Sr, and Zn data, are provided in the sheet labelled “ICPS_analysis” and summarized in Table 4 and Fig. 2. Product yields obtained after HTL at 280°C and 350°C are provided in the sheet labelled “HTL_product_yields” and summarized in Table 5 and Fig. 3. Data on the properties of aqueous products obtained after the HTL reactions are provided in the sheet labelled “Aqueous_phase_analysis” and are summarized in Table 6 and Fig. 4. Data on the biochemical composition of the laboratory and pilot biofilm samples, including protein, ash, lipid, and carbohydrate content, are provided in the sheet labelled “Biochemical_analysis” and are summarized in Table 7 and Fig. 5.

**Table 4:** Mineral compositions data for microalgae biofilm and biochar samples.

	Ca	K	Mg	P	S	Al	As	B	Ba	Cd	Co	Cr	Cu	Fe	Mn	Mo	Na	Ni	Pb	Se	Si	Sr	Zn
	%					mg / kg																	
L_biomass	3.47	0.74	0.76	0.74	0.51	91.0	3.48	26.1	-	-	0.90	1.5	46.3	879	18.6	2.2	4890	1.0	3.0	-	-	236	36.5
P_biomass	5.75 ± 0.03	0.39 ± 0.06	0.67 ± 0.04	4.74 ± 1.12	1.96 ± 1.08	2380 ± 6.42	2.98 ± 4.18	39.4 ± 3.08	505 ± 22.8	3.60 ± 0.35	3.05 ± 0.11	34.0 ± 1.06	381 ± 12.2	19900 ± 771	218.3 ± 4.84	4.7 ± 0.32	5922 ± 388	30.7 ± 0.59	16.6 ± 0.24	3.92 ± 4.66	1700 ± 254	611 ± 10.3	1450 ± 32.7
L_biochar_280	8.22	0.10	1.07	2.94	1.01	1340	0.03	27.15	143	1.00	4.30	24.9	583	7730	138	14.5	1000	34.2	8.8	0.03	2800	542	492
L_biochar_350	24.65	0.08	2.94	7.00	1.02	2730	0.47	6.55	298	2.88	2.80	55.4	912	11900	336	18.7	1060	95.4	23.9	0.03	4960	1520	863
P_biochar_280	12.17 ± 1.37	0.24 ± 0.05	3.44 ± 1.42	9.24 ± 0.90	0.57 ± 0.13	6970 ± 817	1.47 ± 2.50	22.2 ± 1.74	1120 ± 128	8.28 ± 0.86	6.34 ± 0.58	77.6 ± 13.0	805 ± 101	45000 ± 5190	468 ± 41.6	9.4 ± 1.20	4170 ± 2440	67.3 ± 10.1	38.9 ± 4.94	10.2 ± 8.29	4080 ± 1230	1280 ± 139	3040 ± 387
P_biochar_350	12.46 ± 1.33	0.26 ± 0.02	3.95 ± 1.83	9.80 ± 1.56	0.57 ± 0.14	7520 ± 634	2.82 ± 1.67	21.6 ± 1.24	1134 ± 120	8.54 ± 1.28	6.36 ± 0.71	78.7 ± 9.78	830 ± 117	45700 ± 5080	483 ± 29.6	10.7 ± 0.89	4760 ± 162	68.2 ± 9.14	42.1 ± 3.51	12.0 ± 1.56	4670 ± 540	1300 ± 136	3090 ± 372

**Table 5:** Yield in each phase after hydrothermal liquefaction, wt% dry basis.

Feedstock	HTL reaction temperature	Biocrude	Gas	Solid	Aqueous*
L_biomass	280°C	28.7 ± 1.78	7.75 ± 1.32	17.6 ± 3.40	46.0 ± 1.25
L_biomass	350°C	30.0 ± 1.26	9.88 ± 0.50	14.8 ± 3.47	45.3 ± 3.77
P_biomass	280°C	12.7 ± 1.53	14.7 ± 2.32	43.4 ± 2.82	29.2 ± 1.17
P_biomass	350°C	14.0 ± 1.04	16.0 ± 0.11	43.7 ± 4.41	26.3 ± 3.77

⁎ by mass difference

**Table 6:** Properties of aqueous products obtained after HTL reactions.

Sample	pH	Total N (mg / L)	NH3 (mg / L)	TOC (mg / L)	COD (mg / L)
L_AP_280	8.2 ± 0.14	1840 ± 565	848 ± 138	8260 ± 1926	28800 ± 566
L_AP_350	8.4 ± 0.00	4430 ± 25.5	826	6030 ± 25.46	24300 ± 1910
P_AP_280	8.1 ± 0.25	5730 ± 316	1810 ± 335	5820 ± 1461	18500 ± 4660
P_AP_350	8.2 ± 0.06	14600 ± 3280	2320 ± 628	5670 ± 450.4	19800 ± 1270

**Table 7:** Biochemical composition of biofilm samples harvested from laboratory- and pilot-scale RABRs and from trickling filters at CVWRF.

Characteristic (wt%, dry basis)	L_biomass	P_biomass	T_biomass
Protein (N * 6)	36.7 ± 0.97	24.1 ± 0.35	-
Protein (N * 4.78)	29.2 ± 0.77	19.2 ± 0.28	-
Protein (Bradford method)	14.4 ± 3.08	7.93 ± 3.52	12.4 ± 4.27
Extractable lipid content (chloroform/methanol)	7.40 ± 1.22	9.63 ± 0.97	9.66 ± 2.54
Extractable lipid content (hexane/methanol)	-	2.45 ± 0.02	-
Carbohydrate (MBTH/DTT method)	31.4 ± 6.47	10.7 ± 1.27	20.4 ± 6.66
Ash	9.99 ± 1.19	38.6 ± 3.87	18.3 ± 6.22

## 4 Experimental Design, Materials and Methods

### 4.1 Microalgae Feedstock

The biofilm consortium used as the inoculum in this study was collected from trickling filter aeration windows at Central Valley Water Reclamation Facility (CVWRF) in Salt Lake City, Utah. Visual characterization [4] and ongoing 16S/18S/23S/ITS rRNA characterization [2] of this microalgae biofilm consortium have identified community members from *Chlorella, Oscillatoria, Pseudanabaena, Nitzschia, Navicula, Klebsormidium, Ulothrix, Pleurocapsa, Tychonema, Stigeoclonium,* and *Oedogonium,* among others. This consortium was cultivated using an 11,400-liter polyethylene Rotating Algae Biofilm Reactor (RABR) operating in untreated anaerobic digester effluent at CVWRF and in 10-L RABRs under constant artificial lighting (500 µmol/m2/s) at 25°C. This anaerobic digester effluent contains approximately 500 mg/L total Kjeldahl nitrogen (TKN), 50 mg/L phosphorus (TP), and 25 mg/L magnesium. As the 11,400-liter pilot RABR at CVWRF rotates, struvite precipitates from anaerobic digester centrate and collects in the biofilm [4,5]. In a previous work, we observed that phosphorus concentrations in the anaerobic digester effluent used for microalgae cultivation were reduced to 20-30 mg/L after transportation to the laboratory RABRs used in this report [6]. Biomass samples harvested from the pilot RABR at CVWRF for use in this study were collected in spring and winter 2022. All biomass samples collected in this study were harvested by manual scraping and frozen after harvesting.

### 4.2 Biofilm Moisture Content

Moisture content in the biofilm samples was measured by drying samples at 60°C until constant weight [7]. Solids content is reported as *solids content (wt%) = (mass after drying (g) – tare) / (mass before drying (g) – tare)*.

### 4.3 Ash Content

Ash content in biofilm and biocrude samples was measured by incinerating samples at 550°C for 180 min [7]. Briefly, dried biomass samples were heated to 105°C and held for 12 min, ramped to 250°C at 10°C/min and held for 30 min, ramped to 575°C at 20°C/min and held for 180 min, and then cooled to 105°C and held until samples were removed and weighed. Ash content is reported as *ash content (wt%) = (mass after incinerating (g) – tare) / (mass before incinerating (g) – tare)*.

### 4.4 Hydrothermal Liquefaction

HTL experiments were performed in batch mode using a 500 ml stirred-tank pressure reaction chamber (Parr Instruments, Moline, IL, USA) under a nitrogen atmosphere. First, 30 g dry microalgae were resuspended in distilled water (15 % w/w) and added to the pressure chamber at 20°C. The chamber was sealed, sparged and pressurized to 2.0 MPa with nitrogen, and heated at 7°C / min to the pre-selected reaction temperature (280°C or 350°C). After incubation for 15 min at the reaction temperature, the vessel was cooled to 20°C by an internal water coil and the final pressure was recorded. Yield in the gas phase was calculated by pressure difference using the ideal gas law. After venting, the remaining products were rinsed from the pressure vessel with dichloromethane (DCM) and filtered through tared 1.6 µm Whatman filter paper to separate the solid products. After filtration, the biocrude and aqueous phases were separated in a separatory funnel. Yield in the biocrude phase was recorded after vacuum distillation at 40°C for DCM recovery and subsequent drying at 60°C for 12 h. Yield in the aqueous phase was calculated by mass difference. Aqueous products were characterized using HACH kits to measure chemical oxygen demand (HACH method 8000), total nitrogen (HACH method 10208), total ammonia (HACH method 10301), total phosphorus (HACH method 10209/10210), and total organic carbon (HACH method 10267). pH was measured using an Orion 8102BNUWP Ross Ultra Combination pH probe (Thermo Scientific Inc, Waltham, MA, USA).

### 4.5 Elemental Analysis

Elemental analysis (CHNS-O) of the dried biofilm and biocrude samples was conducted using a Flash 2000 CHNS-O analyzer (Thermo Scientific Inc, Waltham, MA, USA) as previously described [8]. Briefly, a five-point calibration curve was generated and validated using a 25-(Bis(5-tert-butyl-2-benzo-oxazol-2-yl) thiophene (BBOT) standard (carbon: 72.53 %, hydrogen: 6.09 %, nitrogen: 6.51 %, oxygen: 7.43 %, and sulfur: 7.44 %). Sample analysis was conducted as follows; 2-3 mg of sample was loaded into a tared tin capsule then loaded into the multi-sample holder. Once the sample was dropped into the quartz reactor (kept at 950°C), the gases produced from its combustion were analyzed using a thermal conductivity detector. Each sample was analyzed in triplicates. The oxygen content was calculated by difference as *Oxygen (wt. %, dry basis) = 100 – Carbon – Hydrogen – Nitrogen – Sulfur – Ash*.

### 4.6 Inductively-Coupled Plasma Spectrophotometry

Analysis of minerals and other elements in the dried biofilm and biochar samples was performed by Utah State University Analytical Laboratories using an inductively-coupled plasma spectrophotometer (Thermo Electron iCAP ICP).

### 4.7 Bomb Calorimetry

Higher heating value (HHV) was measured using an IKA Model C2000 basic bomb calorimeter (IKA Works, Inc., Wilmington, NC, USA). Briefly, 0.5 g samples were loaded into a stainless steel crucible and combusted in a type 2 stainless steel vessel. Acid correction was performed after combustion by titrating bomb washings with a standard sodium carbonate solution.

### 7.8 Protein Content

Protein content was measured using the Bradford assay [9,10] and estimated from elemental compositions data as nitrogen (N) * 4.78 and as N * 6 [11,12]. Biomass samples were digested prior to the Bradford reaction by suspending samples in 0.5 M sodium hydroxide, sonicating for 10 min in a 40 kHz ultrasonic water bath (Branson 1510, Branson Ultrasonics Corp., Danbury, CT, USA), incubating at 80°C for an additional 10 min, and then centrifuging to remove any residual biomass. Protein concentration in the supernatant was estimated by reacting 50 µL sample with 150 µL Bradford Reagent for 5 min and comparing absorbance at 595 nm to a bovine serum albumin standard.

### 4.9 Lipid Content

Lipid content was measured using a colorimetric method based on the sulpho-phospho-vanillin (SPV) reaction [13] and gravimetrically after extraction with methanol and hexane [14,15]*.* In the SPV method, lipids were first extracted from dry biomass samples using a 2:1 v/v chloroform: methanol extraction. This reaction was performed at room temperature with an extraction ratio of 1 mg dry biomass: 2 ml solvent: 1.5 ml 0.9 % NaCl solution. After the extraction, 0.5 ml of the chloroform/lipid phase was transferred to a clean test tube, heated uncapped at 90°C to evaporate the chloroform, and reacted with 100 µL of 98 % sulfuric acid at 90°C for 10 min. After this reaction, samples were cooled to room temperature, mixed with 2.4 ml of SPV reagent, and incubated at room temperature for another 10 min to allow color development before measuring absorbance at 530 nm. Lipid content was estimated by comparing samples to a standard curve prepared using canola oil. The SPV reagent used in this reaction was prepared by dissolving 750 mg vanillin in 125 ml DI water and 500 ml 85 % phosphoric acid.

To quantify hexane/methanol extractable lipids, lipids were first extracted by sonicating 100 mg dry algae in 1 ml of 1:1 v/v hexane: methanol for 15 min at 40 kHz and then incubating at room temperature for 1 h and 45 min in a rocking incubator. After incubating, samples were centrifuged for 10 min at 12,000 x g and the solvent layer was transferred to a clean tube. Distilled water was added to separate the methanol and hexane layers, and the upper hexane/lipid layer was aspirated into a clean pan and heated at 60°C to evaporate the hexane before weighing. Hexane extractable lipids were recorded as hexane extractable lipids (wt%) = mass in hexane phase (mg) / original mass (mg).

### 4.10 Carbohydrate Content

Carbohydrates were measured using a colorimetric 3-methyl-2-benzothiazolinone hydrazone (MBTH) / dithiothreitol (DTT) assay [16]. First, MBTH and DTT stock solutions were prepared by dissolving 30 mg MBTH in 10 mL distilled water and 10 mg DTT in 10 mL distilled water, respectively. MBTH/DTT reagent was prepared immediately before sample analysis by mixing 5 mL MBTH stock solution with 5 mL DTT stock solution. Ferric reagent was prepared by dissolving 200 mg ferric ammonium sulfate and 200 mg sulfamic acid in 40 mL of 0.25 M hydrochloric acid. Before the colorimetric reaction, 25 mg dry biomass samples were hydrolyzed in 250 µL of 72 % sulfuric acid at 30°C for one hour. After this hydrolysis, samples were diluted by the addition of 7 mL distilled water, autoclaved at 121°C for one hour, and filtered through 0.2 µm nylon syringe filters. Samples were further diluted by distilled water as necessary for the colorimetric reaction. For the colorimetric reaction, 50 µL of hydrolyzed sample was vortexed with 500 µL 0.5 M sodium hydroxide, 500 µL MBTH/DTT reagent, and 450 µL distilled water and then incubated in a pre-heated 80°C heat block for 15 min. After 15 min, 1 mL of ferric reagent was added to each tube in the heat block. Next, samples were removed from the heat block, vortexed, and allowed to cool for 10 min. A final volume of 2.5 mL distilled water was added to each sample immediately after the 10-min cooling period. Carbohydrate content was estimated for these samples by measuring absorbance at 620 nm and comparing to a glucose standard curve.

## Limitations

Not applicable.

## Ethics Statement

The authors have read and follow the ethical requirements for publication in Data in Brief and confirm that the current work does not involve human subjects, animal experiments, or any data collected from social media platforms.

## CRediT authorship contribution statement

**Jacob D. Watkins:** Conceptualization, Methodology, Validation, Formal analysis, Investigation, Data curation, Writing – original draft, Writing – review & editing, Visualization, Supervision, Project administration. **Hamza Abdellaoui:** Methodology, Validation, Investigation, Writing – original draft. **Elise Barton:** Methodology, Investigation. **Clayton Lords:** Methodology, Investigation. **Ronald C. Sims:** Conceptualization, Methodology, Resources, Writing – review & editing, Supervision, Funding acquisition.

## Data Availability

Mendeley DataDataset on biochemical composition and hydrothermal liquefaction of a native microalgae biofilm grown using municipal anaerobic digester effluent in Salt Lake City, Utah (Original data).
